# Graphene oxide-dependent growth and self-aggregation into a hydrogel complex of exoelectrogenic bacteria

**DOI:** 10.1038/srep21867

**Published:** 2016-02-22

**Authors:** Naoko Yoshida, Yasushi Miyata, Kasumi Doi, Yuko Goto, Yuji Nagao, Ryugo Tero, Akira Hiraishi

**Affiliations:** 1 Center for Fostering Young and Innovative Researchers, Nagoya Institute of Technology, Nagoya, Aichi 466-8555, Japan; 2 Electronics-Inspired Interdisciplinary Research Institute (EIIRIS), Toyohashi University of Technology, Toyohashi, Aichi 441-8580, Japan; 3 Nagoya Municipal Industrial Research Institute, Nagoya, Aichi 456-0058, Japan; 4 Department of Civil Engineering, Nagoya Institute of Technology, Nagoya, Aichi 466-8555, Japan; 5 Department of Biomedical Science, College of Life and Health Science, Chubu University, Kasugai, Aichi 487-8501, Japan; 6 Department of Environmental and Life Sciences, Toyohashi University of Technology, Toyohashi, Aichi 441-8580, Japan

## Abstract

Graphene oxide (GO) is reduced by certain exoelectrogenic bacteria, but its effects on bacterial growth and metabolism are a controversial issue. This study aimed to determine whether GO functions as the terminal electron acceptor to allow specific growth of and electricity production by exoelectrogenic bacteria. Cultivation of environmental samples with GO and acetate as the sole substrate could specifically enrich exoelectrogenic bacteria with *Geobacter* species predominating (51–68% of the total populations). Interestingly, bacteria in these cultures self-aggregated into a conductive hydrogel complex together with biologically reduced GO (rGO). A novel GO-respiring bacterium designated *Geobacter* sp. strain R4 was isolated from this hydrogel complex. This organism exhibited stable electricity production at >1000 μA/cm^3^ (at 200 mV vs Ag/AgCl) for more than 60 d via rGO while temporary electricity production using graphite felt. The better electricity production depends upon the characteristics of rGO such as a large surface area for biofilm growth, greater capacitance, and smaller internal resistance. This is the first report to demonstrate GO-dependent growth of exoelectrogenic bacteria while forming a conductive hydrogel complex with rGO. The simple put-and-wait process leading to the formation of hydrogel complexes of rGO and exoelectrogens will enable wider applications of GO to bioelectrochemical systems.

Bioelectrochemical systems (BESs)^1^ or microbial electrochemical systems (MESs)^2^ are the devices of electrochemical reactions using microorganisms as the catalysts. Microbial fuel cells (MFCs) are a representative of BESs that generates electrons to an electrode via microbial oxidization of organic compounds^3^. Exoelectrogenic bacteria are characterized by their unique function called extracellular electron transfer (EET)^4^ and are mediators in electricity production in BESs. Members of the genera *Geobacter* and *Shewanella* are the most studied exoelectrogens that can transfer electrons directly by attachment to the electrode and indirectly via redox mediators^5^,^6^.

The performance of BESs is associated mainly with EET in bacterial biofilms developing on electrode but less with indirect EET by planktonic cells within the apparatus^7^. Therefore, electrode material is an important determinant of the formation of biofilms and the performance of electron transfer on the cell-electrode interface. Since carbon electrodes are chemically stable and good for development of bacterial biofilms, this type of electrodes has preferably been applied to BESs^8^,^9^. Especially, graphite felt, carbon brush, and carbon cloth have been taken into account for practical use because of their commercial availability, experimental performance, and economic benefit. One of the possible important factors affecting the performance of electrodes is the surface area. An electrode having a larger surface area may allow the attachment of more bacterial cells than that of bare carbon or graphite^10^. A recent technical progress in this research area is the modification of anode by using graphene derivatives that exhibit higher performance^11^,^12^,^13^.

Graphene, a single layered honeycomb lattice of carbon atom, has advantages of having high conductivity and large surface areas at different order magnitude, e.g. 2965 m^2^/g for graphene^14^ and 0.02 m^2^/g for graphite felt^10^. However, it is difficult to apply graphene directly to BESs, because it is of hydrophobic powder and is required to be a complex with supportive electrodes for use in BESs. It has been shown that the addition of graphene oxide (GO), the oxidized form of graphene, to the reaction chamber in BESs enhances electron transfer to carbon electrode^15^,^16^. GO itself is not electrically conductive but becomes a conductor when reduced by microorganisms^17^,^18^. Here this reduced form of GO is designated simply as reduced GO (rGO), because no information on its chemical identity is available. Bacterial reduction of GO was first demonstrated in cultures of *Shewanella* species and later in *Escherichia coli*^19^ and complex mixed populations^20^. *Shewanella* species reduced GO by using redox protein involved in EET^17^,^18^. Also, GO was reduced in the cell-free extract from a *Shewanella* culture^17^, possibly by small biomolecules like vitamin C^21^. These findings raise a question of whether GO serves as an electron acceptor for EET coupling with oxidation of the substrate in exoelectrogenic bacteria, thereby allowing their growth. So far, bacterial growth by GO respiration has not yet been fully demonstrated. On the other hand, GO has also been shown to have antibacterial or bactericidal activities^22^,^23^. These results provide another assumption that GO works as a simple electron sink but not as a terminal electron acceptor to allow respiratory growth. Therefore, information on the effects of GO on bacterial growth and metabolism is fragmentary and indefinite at present. This situation has restricted the application of GO to BESs.

GO has potentially greater advantages than graphene in application to BESs, partly because GO is more economic than graphene, and partly because the hydrophilicity of GO may allow the attachment of more bacterial cells to its surface in aqueous solutions. If GO can function as a terminal electron acceptor supporting growth of exoelectrogenic bacteria, GO may be useful for selective enrichment of these bacteria from the environment. Also, it is of great interest to note that GO self-aggregated into a hydrogel when chemically reduced in aqueous solutions^24^. This makes us expect that GO is usable for the formation of a high-dense assembling complex of EET-driving exoelectrogens and conductive rGO.

The main purpose of this study was to determine whether selective growth and self-aggregation of exoelectrogenic bacteria take place depending upon GO. For this purpose, we attempted to make enrichment cultures of GO-respiring bacteria (GORBs) from the environment and examined these GORBs for electricity production via EET to electrode. The phylogenetic composition of the enriched GORBs was determined by high-throughput sequencing of 16S rRNA gene amplicons. As reported herein, our attempts to make a conductive hydrogel complex of GORBs and rGO gave positive results. To the best of our knowledge, this study is the first to demonstrate GO-dependent growth of exoelectrogenic bacteria accompanied with self-aggregation into a hydrogel complex with rGO that is capable of electricity production.

## Results

### Characterization of GO

GO samples used in this study were commercially obtained as powder and dispersed in water by sonication for 2 h. The prepared GO almost consisted of single-layered sheets with 1.5 nm thickness as seen in AFM images, and had abundant C = O peaks at 287 eV and a C-C/C-H peak at 285 eV in XPS (Supplementary Fig. S1). The area of GO sheets was variable with an average of 0.26 ± 0.46 μm.

### Growth of GORBs in enrichment cultures

To enrich GORBs from environmental samples, acetate was used as the sole carbon and energy source. The redox potentials of acetate and a chemically reduced GO were −290 mV^25^ and +380 mV^26^ vs SHE (standard hydrogen electrode), respectively. The difference in the reduction potential between the two is thermodynamically favorable for the generation of energy and the resultant growth by respiratory GO reduction coupling with acetate oxidization.

The enrichment of GORBs was performed using three different freshwater environmental samples as the seed, i.e., river water (RW), paddy soil (PS), and water channel sediment (WC). These samples were incubated with GO and acetate for several days. Then, portions of the cultures were transferred to fresh medium to continue the cultivation. By repeated transfer and cultivation of more than 10 times, we obtained highly enriched cultures designated as cultures RW, WC, and PS.

**Figure 1:**
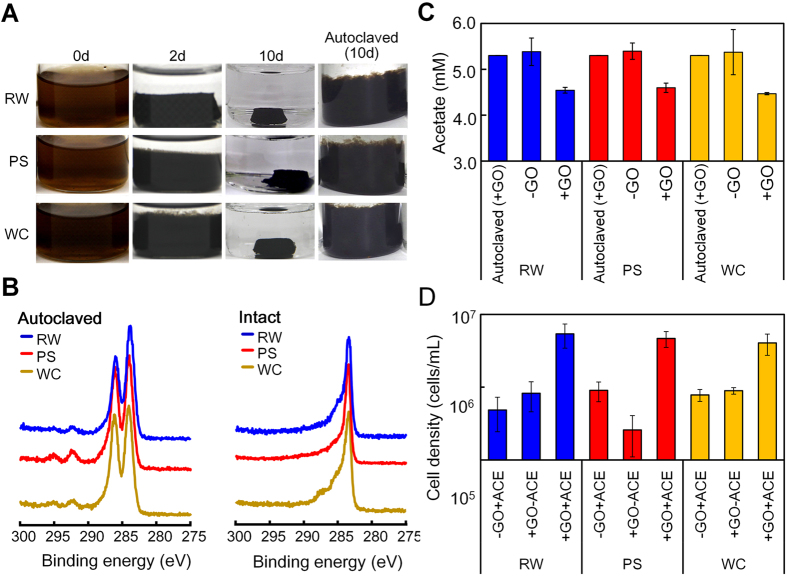
GO-dependent growth and self-aggregation of microbial cells in enrichment cultures. (**A**) Appearance of the three enrichment cultures (cultures-RW, -PS, -WC) inoculated with autoclaved and intact cells, showing the formation of a self-aggregated hydrogel complex. (**B**) XPS data of GO and the partially reduced GO in the cultures with autoclaved or intact inocula after 10 days. (**C**) Acetate concentration in the cultures with and without GO after 10 days. (**D**) Cell density in the cultures with and without GO and acetate (ACE) after 10days.

At the beginning of the enrichment process, the added GO was dispersed entirely in the cultures, resulting in brown suspensions (Fig. 1A). Then, the cultures gradually turned black and formed a dense hydrogel complex during a 10 days-period of incubation. An XPS analysis showed that the GO in the three cultures had C1s spectra with respective two major peaks approximately at 287 eV for C = O and 285 eV for C-C/C-H bonds at 0 d (Fig. 1B). The C = O peak initially dominated and lowered gradually with time in all cultures. Meanwhile, the C-C/C-H peak became dominant and the C = O peak almost disappeared. No or little changes in appearance and the XPS peak were observed in the autoclaved cultures as the control (Fig. 1A,B). These observations indicated that the reduction of GO as judged by the change in color from brown to black depends upon microbial activity.

In the three enrichment cultures, acetate was consumed along with the reduction of GO but remained unchanged in the control without GO (Fig. 1C). The simultaneous decrease in the C = O peak intensity and the acetate concentration suggested that GO reduction coupled with acetate oxidization. When the hydrogel complex was dispersed in the aqueous phase by homogenization, the cell density was approximately 20-fold higher in the GO- and acetate-amended cultures than in the cultures without either additive or both, accounting for (4.0–7.6) × 10^6^ cells/mL (Fig. 1D). Given the fact that the present bacteria required both GO and acetate for growth, it is logical to conclude that this growth actually depends upon anaerobic respiration with GO as the terminal electron acceptor and acetate as the carbon and energy source.

**Figure 2:**
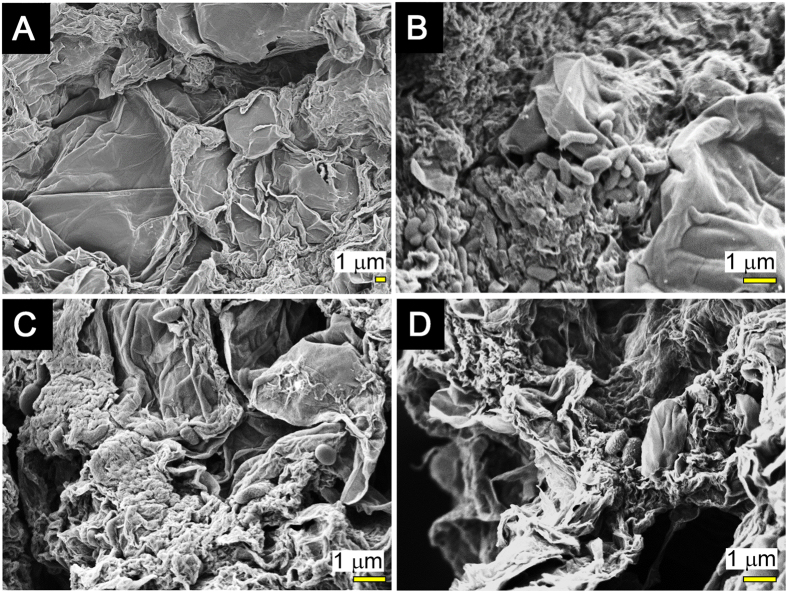
SEM images of GO and the rGO-GORBs complexes. (**A**) SEM image of GO used in this study. (**B–D**) SEM images of the rGO-RGOBs complexes in culture-RW (**B**), -PS (**C**), -WC (**D**), respectively.

In the enrichment cultures with GO, 80 ±12% of cells were distributed in the developed hydrogel complex (Table 1). SEM imaging of the hydrogel complex revealed that the inclusion of bacterial cellular structures occurred on the surface of the complex (Fig. 2). The morphotypes of bacteria in the three cultures were similar to one another, as curved rods were abundant and cocci were occasionally observed. These cell structures were not observed on GO itself before incubation.

### Phylogenetic identification of GORBs in the complex

**Figure 3:**
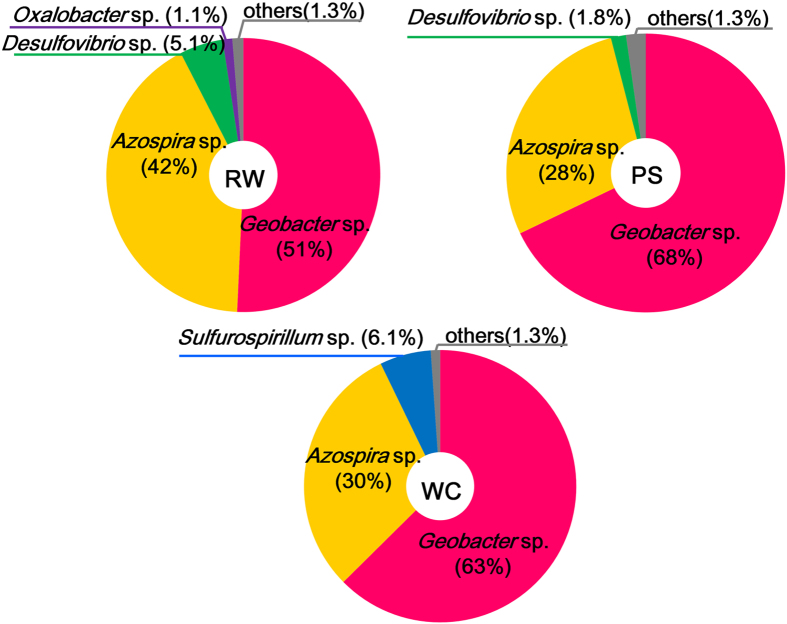
Identification of bacteria grown with GO. The pie charts of phylogenetic identification of operational taxonomic units (OTUs) in three cultures (culture-RW, -PS, –WC). The OTUs having more than 1% frequency were shown in the charts.

To identify bacteria in the black hydrogel complexes of rGO and GORBs, 16S rRNA genes from these complexes were PCR-amplified and analyzed by high-throughput sequencing with the Illumina MiSeq platform. In all three cultures, the 16S rRNA genes of *Geobacter* species, well-known exoelectrogenic bacteria, were most abundant, accounting for 51–68% of the total amplicons (Fig. 3). Beside the *Geobacter* bacteria, members of *Azospira* were significantly detected in all three cultures, comprising 28–42% of the total population. *Azospira* species, which are acetate oxidizers, have been detected frequently in the anodic chamber of MFCs^27^,^28^.

### Electrochemical cultivation of the rGO-GORBs complexes

**Figure 4:**
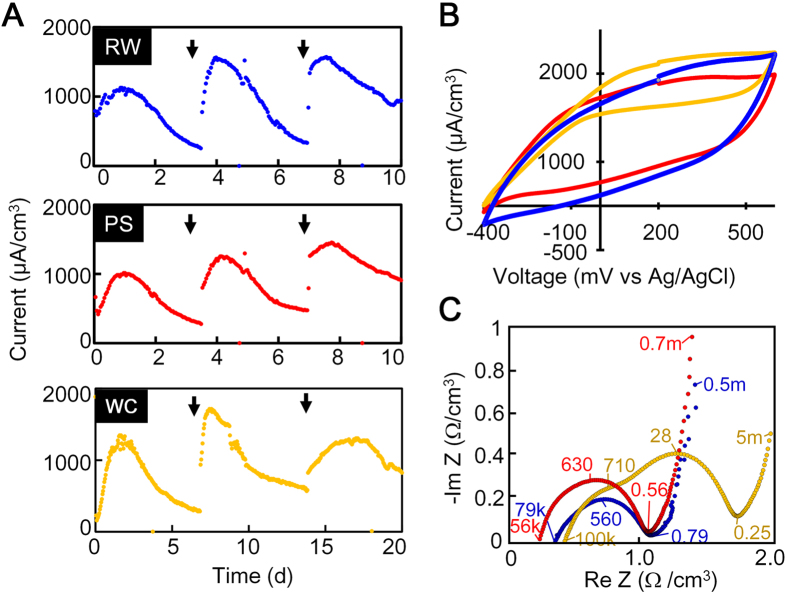
Electricity production by the rGO-GORBs complexes. (**A**) Changes in electricity produced by the rGO-GORBs complexes inoculated with RW, PS, and WC. (**B**) CV obtained by the three rGO-GORBs complexes. (**C**) EIS obtained by the three rGO-GORBs complexes. The numbers shown in graph are frequencies: f [Hz] = ω/2π.

As noted above, we successfully obtained the rGO-GORB hydrogel complexes through the enrichment process with GO and acetate. To ensure the ability of these complexes to convert acetate to electricity, they were polarized at +200 mV (v.s. Ag/AgCl). Electricity in all three complexes was rapidly generated with the maximum level appearing in a range of 1,000–1,300 μA/cm^3^ on days 1–2 (Fig. 4A). The electricity production decreased gradually with time but was recovered upon spiking with acetate. The immediate and stable production of electricity was due to significant growth and stable activity of GO-reducing exoelectrogens in the complexes after polarization. After 10–20 days of the polarization with the triple addition of 5.0 mM acetate, the total cell counts in the three cultures ranged from (3.7–4.9) × 10^9^ to (0.53–3.8) × 10^12^ cells/culture (Table 1), and 64–89% were present within the complexes. The cell density per unit volume was 160-fold higher (0.5 × 10^11^ to 3.1 × 10^11^ cells/cm^3^) in the complexes than in the liquid phase with planktonic cells (1.7 × 10^8^ to 5.3 × 10^8^ cells/mL). In all the complexes, *Geobacter* species constituted overwhelming majorities (77–91%) of the electrochemically grown populations (Supplemental Table S1).

The CV curves for the three rGO-GORB complexes showed the catalytic current of acetate oxidization, e.g., 1200–1700 μA/cm^3^ at 500 mV v.s. Ag/AgCl with 0.2 mV/s of scan rate (Fig. 4B). No obvious redox peak appeared. The voltammograms for cultures RW and PS showed inflated symmetric discharges of current, indicating that the electric double layer capacitance depended upon the large surface area of the rGO-GORBs complexes. In the WC culture, the CV curve became skewed at lower potentials. On the basis of the Nyquist plot using raw data for the three complexes (Fig. 4C), the charge transfer resistances (*R*_ct_), expressed as the diameter of semicircles, were <1.0 Ω/cm^3^ in cultures RW and PS and 1.0–2.0 Ω/cm^3^ in culture WC. The two apparent semi-circles in WC indicated that two electrochemical reactions were involved. The smaller semicircle was similar to those observed in the RW and PS cultures, suggesting that an electrochemical kinetic reaction by *Geobacter* took place. The larger semicircle possibly showed the involvement of electrochemical kinetic reactions by other bacteria such as *Sulfurospirillum* species certainly grown in WC (13% of total population) but not or less grown (<0.1%) in RW and PS (Supplementary Table S1).

### Growth of GO-respiring *Geobacter* sp. in pure culture

**Figure 5:**
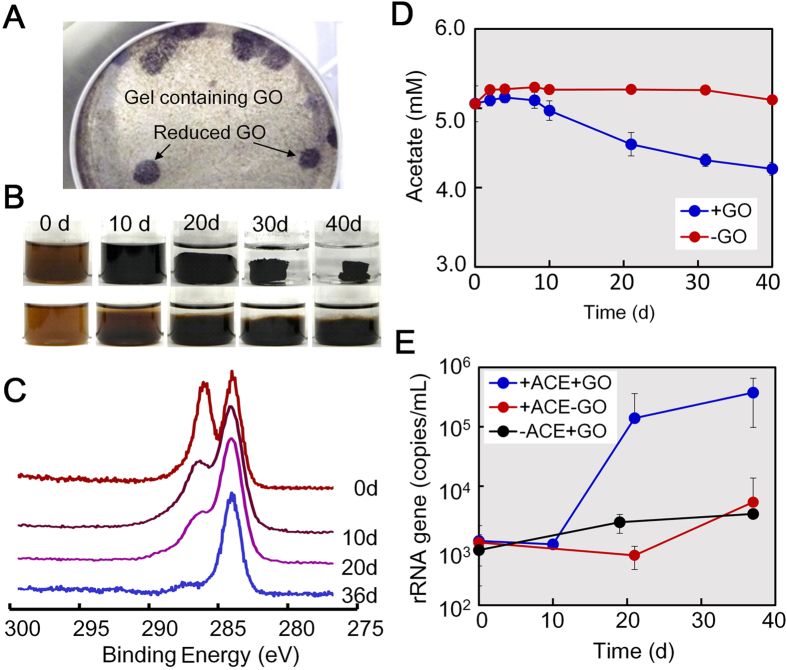
GO-dependent growth and self-aggregation of *Geobacter* sp. strain R4 cells. (**A**) Black halo formation by strain R4 in an agar plate as being positive for GO reduction. (**B**) GO reduction and self-aggregation of cells into a hydrogel complex in the R4 cultures inoculated with intact (upper) and autoclaved (lower) inocula. (**C**) XPS data of C1s spectra in the intact R4 culture. (**D**) Acetate concentration in the intact R4 cultures with and without GO. (**E**) The number of 16S rRNA gene copies in the intact R4 cultures with and without GO and acetate (ACE).

To determine whether the enriched *Geobacter* bacteria are actually able to grow by GO respiration, attempts to isolate *Geobacter* from the enrichment cultures were made by anaerobic agar cultivation with GO. As the results, the RW culture yielded small colonies with a large black halo in the agar medium as being positive for GO reduction (Fig. 5A). A single colony from this agar plate was successfully purified by repeated agar dilution and designed as strain R4. By 16S rRNA gene sequencing, strain R4 was identified actually as a member of the genus *Geobacter* with *Geobacter bremensis* strain Df1^T^ (accession number U96917) as its closest relative at a 99.3% level of similarity.

A pure culture of *Geobacter* sp. strain R4 was capable of reducing GO and forming a black hydrogel complex with rGO (rGO-R4 complex) similar to that seen in culture RW (Fig. 5B,C). Strain R4 required 30 d to form the rGO-R4 complex, while it took 10 d to form the rGO-RW complex in the mixed culture. Anaerobic growth of strain R4 occurred along with acetate consumption and GO reduction (Fig. 5D,E). In an XPS analysis of three samples taken from different positions of the complex on day 36, the abundant C-C bonds as main C1s peak was repeatedly observed (Supplementary Figure S2), suggesting that GO in the complex was all over reduced. The electric conductivity of the rGO-R4 complex was 16 mS/cm, which is much higher than that recorded for the original GO via microbial reduction (Supplementary Figure S3A,B).

These results show that *Geobacter* sp. strain R4 is able to grow anaerobically by GO respiration without any association with other microorganisms and to form a conductive hydrogel with rGO.

### Electricity production in the rGO-R4 complex

**Figure 6:**
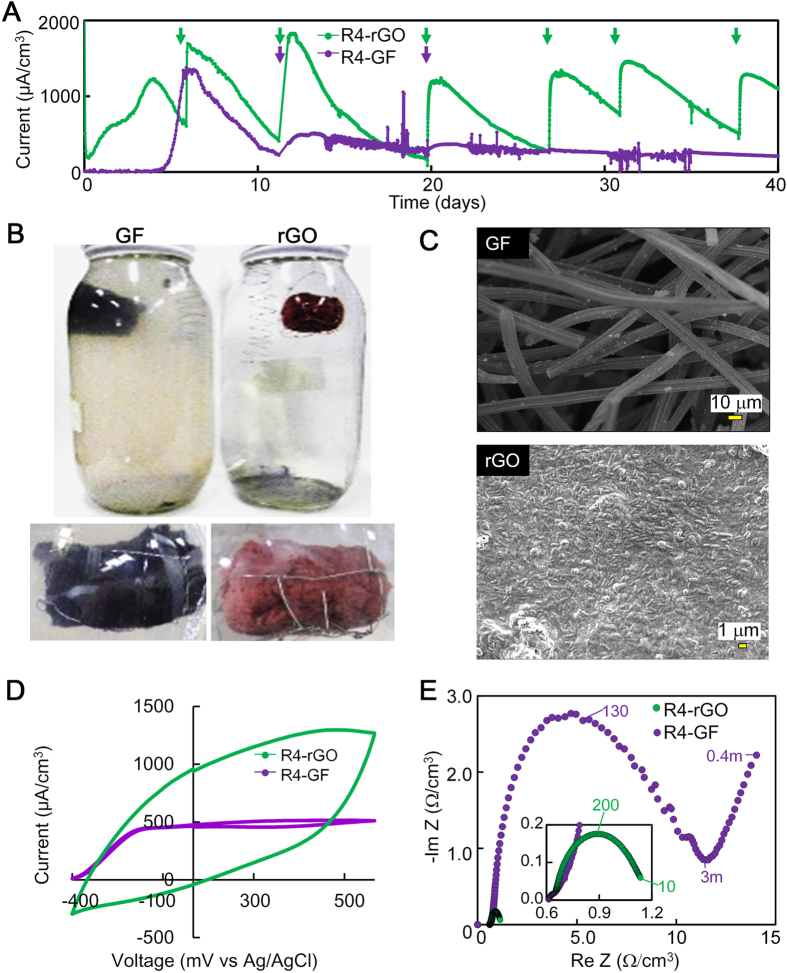
Electricity production by *Geobacter* sp. strain R4 using GO and graphite felt as the anode. (**A**) Changes in electricity in the rGO-R4 (green) and GF-R4 (purple) complexes. (**B**) Appearance of the rGO-R4 and GF-R4 complexes after 30 days of incubation. (**C**) SEM image of the rGO-R4 and GF-R4 complexes after 30 days of incubation. (**D**) CV curves obtained by the rGO-R4 and GF-R4 complexes. (**E**) EIS data obtained by the rGO-R4 and GF-R4 complexes. The inset shows the enlarged same data in narrow range. The numbers shown in graph are frequencies: f [Hz] = ω/2π.

The rGO-R4 complex was examined for its ability to produce electricity. For comparison, graphite felt (GF), a representative anode widely used in MFCs, was used. In both the complex systems, the number of cells present were approximately 8.0 × 10^8^ cells/complex. Cells of strain R4 produced electricity in both complexes at 1,300–1,700 μA/cm^3^ within 7 d but exhibited different production profiles depending upon anodes used (Fig. 6A). Electricity in the GF-R4 complex was gradually reduced with time and not recovered by spiking with acetate, while electricity in the rGO-R4 complex was stably generated. These results were highly reproduced in repeated assays as shown in Supplementary Fig. S2. The concentration of acetate in the rGO-R4 culture became lower than the detection limit after 20 d of incubation. On the other hand, more than half of added acetate (8.0 mM) still remained in the GF-R4 culture (Supplementary Figure S4). These results indicate that rGO-R4 culture can oxidize acetate and generate electricity much more efficiently than the GF-R4 culture.

The rGO-R4 complexes turned pinkish after one week of polarization (Fig. 6B). This time-dependent coloring was similar to that seen typically in the multilayered biofilm of *Geobacter*. The density of cells in the rGO-R4 complex changed from 8.0 × 10^8^ cells/cm^3^ to 3.6 × 10^10^ cells/cm^3^, accounting for 93% of the total population in the whole culture (Table 2). SEM observation indicated the surface of the rGO-R4 complex was indeed covered with abundant cells (Fig. 6C). All of these results suggested the formation of multilayered biofilm on the rGO-R4 complex by electrical polarization. In contrast, in the GF-R4 culture, no apparent biofilm was developed but a pink turbid liquid phase was formed (Fig. 6B). The total mass of cells in the GF-R4 culture was no more than 6.4% of that in the rGO-R4 culture (Table 2) and 64% of the cells were planktonic. This lower level of biomass might be related to the electrochemical and physiological profiles in the GF-R4 culture.

To evaluate the effect of hydrogel structure of the rGO-R4 complex on electricity production, strain R4 was cultivated electrically using graphite coated with or without GO (Supplementary Figures S5 and S6). In both cultures, strain R4 formed biofilms on the surface of graphite (Supplementary Table S3) and produced electricity. Unlike the case in the hydrogel complex, however, the electricity production in both cultures declined gradually with time. These results indicate that the hydrogel structure is important to maintain the electricity production by strain R4.

The CV analysis of the rGO-R4 complex after polarization showed a much higher catalytic current (1,300 μA/cm^3^) in the rGO-R4 complex at 500 mV (vs Ag/AgCl) than in the GF-R4 complex (470 μA/cm^3^) (Fig. 6D). The greater production of electricity with rGO was probably due to larger capacitance and much smaller resistance than those noted with GF (Fig. 6E). EIS data clearly showed 10-fold smaller *R*_ct_ in the rGO-R4 than in GF-R4 complex, i.e.,<1.0 Ω/cm^3^ and >10 Ω/cm^3^, respectively. In ideal electrochemical kinetic reactions, the capacitance (C) is inversely proportional to *R*_ct_ and the angular frequency (ω_max_) showing the top of semicircle (ω_max_CR_ct_ = 1). Hence, the capacitance in rGO-R4 can be estimated to be greater than that of GF-R4.

A better performance of the rGO-R4 complex was also observed even before electric polarization (Supplementary Figure S7). The results clearly indicated that the rGO-R4 complex has originally had electric double layer capacitance and small charge transfer resistance before the formation of biofilm. The biofilm formed on the complex after polarization showed 0.46 mS/cm of electric conductivity, which is much lower than the value for the rGO complex (16 mS/cm). These results indicated the greater electric capacitance and smaller charge transfer resistance were provided from the hydrogel complex itself rather than biofilm on the complex, although potentially supported partly from biofilm.

## Discussion

Although GO is a nanomaterial having great promise in application to BESs, it has been in dispute in connection with its effects on bacterial growth and metabolism. During the past decade, a number of studies have shown that GO has antibacterial or bactericidal effects on a wide variety of species^22^,^23^,^29^,^30^. Thus, the main objective of this study was to determine whether GO functions as the terminal electron acceptor to support growth of exoelectrogenic bacteria, and our attempt to demonstrate this was successful. The GO-dependent-growth allowed selective enrichment of exoelectrogenic bacteria in the complex (Figs 1 and 3). The use of this process has a greater advantage to reduce GO than other abiotic GO reduction techniques. The inconsistent results of the previous studies showing antibacterial activity and ours probably arise from differences in experimental conditions with GO set up. Recent articles have indicated that antibacterial activity of GO is detectable only on the surface coated with small-sized GO but not in the aqueous solution^31^,^32^. Therefore, the growth of exoelectrogenic bacteria we found may be induced by the non-cytotoxic state of GO in aqueous solution^31^.

One of the most striking observations in this study is that the cultivation of environmental samples resulted in the formation of self-aggregated hydrogel complexes of exoelectrogenic bacteria and rGO which can work as a conductor. The mechanism of this hydrogel formation is unknown with certainty, but this is possibly caused by partial π-π stacking of the rGO, as previously observed in the hydrothermal reduction of GO to the rGO hydrogel^24^. Therefore, the formation of rGO hydrogel probably depends on the bacterial ability to reduce GO. When *Shewanella* species^17^,^18^and *Escherichia coli*^19^ were cultivated with GO, the resultant rGO was present as flocs but not as a hydrogel complex. Nevertheless, these bacterial species may form the rGO hydrogel under the same cultural conditions as used in this study. Thus, the present study provides a new model of bio-conductors as a high-dense assembly of rGO and bacteria.

As reported herein, the *Geobacter* populations enriched with GO from the environment into the hydrogel complexes are much higher than those detected in natural environments such as paddy soil^33^, wetland sediment^34^, and aquifer sediment^35^. It is also worthwhile noting that the rGO-GORBs complexes included higher *Geobacter* populations than MFC fed with acetate, where *Geobacter* constituted 14–60% of the total population^36^,^37^,^38^,^39^. This suggests that the cultivation using GO is more useful for the selective enrichment of *Geobacter* and other EET-driving bacteria compared to the conventional system under electric polarization. Besides *Geobacter* species, members of *Azospira* were detected at significant proportions (28–41% in total) in the enrichment cultures. Although *Azospira* species have been frequently detected in MFC^27^,^28^, none of them have been reported to reduce extracellular electron acceptors. One of the characteristics features of EET-driving bacteria, such as *Geobacter* and *Shewanella* species^40^, is the ability to oxidize humic substances, and similarly, an *Azospira* strain has been reported to oxidize humic substances^41^. Based on these collective results, one can assume that the *Azospira* bacteria in the rGO-GORBs complexes are involved in GO reduction. In culture WC, the proportion of *Sulfurospirillum* increased from 6.1% to 13% by polarization. In view of this, together with the fact that members of *Sulfurospirillum* are capable of EET to iron oxides^42^, these bacteria may play a role in electricity production in the rGO-GORBs complex.

*Geobacter* sp. strain R4, a newly isolated bacterium in this study, exhibited biofilm growth and stable electricity production with rGO while showing planktonic growth and temporary electricity production with GF (Fig. 6). This difference in the bioelectrochemical features between the two cultures suggests that strain R4 changes the mode of EET depending upon carbon materials, i.e., direct EET to rGO and indirect EET to GF via aqueous redox molecules. It has been shown that, in exoelectrogenic *Geobacter* species, the direct EET are catalyzed by outer membrane proteins including *c*-type cytochromes and conductive bio-filament. Planktonic cell growth of *Shewanella oneidensis*^43^ under polarization is associated to produce electron shuttles such as flavins^44^. It is notable that the electrode-dependent switching of the EET mode by a pure culture was firstly found in this study. The unique characteristics of *Geobacter* sp. strain R4 will provide a new insight into comprehensive understanding of the interaction of exoelectrogenic bacteria with carbon electrode.

To the best of our knowledge, this study is the first to demonstrate GO-dependent selective growth of exoelectrogenic bacteria and the formation of a self-aggregated hydrogel complex of the biomass and rGO. The rGO-GORB complex has better biofilm growth, much smaller internal resistance and larger capacitance than does the GF-used system. The simple put-and-wait process leading to self-aggregation into the rGO-biomass complex and the enhancement of EET between bacteria and the electrode will contribute to the expansion of the application of GO in BESs.

## Methods

### Preparation and AFM imaging of GO

GO powder was purchased from Royal Elite New Energy Science & Technology Co., Ltd (Shanghai, China). GO was dissolved in sterilized MilliQ water to give a concentration of 10 g/L, sonicated for more than 2 h using a Bransonic model CPX sonicator (Branson Ultrasonics, Emerson Japan, Ltd., Atsugi, Japan), and stored at 4 °C until use. The GO thus prepared was analyzed for thickness and flake area with an Agilent PicoPlus 5500 atomic force microscope (Agilent Tech. Inc., Santa Clara, CA).

### XPS analysis

Chemical states of GO and biologically reduced GO (rGO) were analyzed by X-ray photoelectron spectroscopy (XPS). For this analysis, the GO and rGO were deposited and dried on a silicon wafer. The silicon wafers were collected, washed several times with MilliQ water, and dried as described previously^17^. XPS spectra were measured using an XPS instrument, Versa Probe PHI-5000 (ULVAC-PHI Inc., Osaka, Japan) equipped with a monochromatic Al Ka X-ray source and operated at a bare pressure lower than 10^−6^ Pa. The core level spectrum of C1s was obtained through focused scans after survey scanning over 0–1,100 eV.

### Enrichment and isolation of GO-respiring bacteria

GORBs were enriched by anaerobic cultivation of environmental samples using a chemically defined medium with acetate as the carbon and energy source and GO as the electron acceptor. As the seed, we used three different environmental samples, i.e., river water (designed RW, 34°42′22″N, 137°23′31″E), sediment in a water channel (designed WC, 34°42′26″N, 137°23′42″E), and a paddy soil (designed PS, 34°42′37″N, 137°23′47″E). The three cultures RW, WC, and PS were independently incubated and sequentially transferred to fresh medium periodically as described in supporting information. Subsequent to the enrichment, GORBs from the enrichment cultures were isolated by the agar dilution technique as described in supporting information.

### Phylogenetic identification of enriched and isolated GORBs

Enriched and isolated GORBs were phylogenetically identified by sequencing of 16S rRNA genes. 16S rRNA genes from the enrichment cultures were PCR-amplified with the bulk DNA as the template and sequenced using the Illumina MiSeq system as described in supporting information. A GORB isolate from the enrichment culture was subjected to PCR amplification of 16S rRNA gene using primers 27f and 1492r (supporting Table 1). The 16S rRNA gene amplicon was sequenced using an Applied Biosystems Big Dye Terminator V3.1 kit (Life Technologies, Carlsbad, CA) and an Applied Biosystems 3730xl DNA Analyzer (Life Technologies) as described previously^45^.

### HPLC analysis and determination of cell density

For quantification of acetate in the culture, a part of the culture was taken and analyzed using a reverse-phase HPLC system (Shimadzu, Kyoto, Japan) equipped with L-column2 ODS (4.6 × 250 mm) (CERI, Tokyo, Japan) and a UV detector as described previously^46^. Direct total cell counts in the enrichment cultures, RW, PS, and WC were determined by epifluorescence microscopy with SYBR GREEN II staining^47^. To monitor cell growth in a pure culture of strain R4, real-time qPCR was conducted by using a universal primer set for the bacterial 16S rRNA genes, 357f and 517r (supplementary Table S2). The qPCR was performed with a LightCycler FastStart DNA Master SYBR Green I kit (Roche Molecular Biochemicals, Indianapolis, IN) and the LightCycler Nano system (Roche Applied Science, Indianapolis, IN) as described previously^45^. As the standard, the purified 16S rRNA gene amplicon from genomic DNA of strain R4 was used.

### Preparation of the rGO-GORBs complex

For assays of microbial electricity production in the rGO-GORBs complexes, enriched and pure cultures of GORBs were incubated in a glass bottle of 0.93 L capacity (size, 90 mm diameter and 175 mm height) with the following minor modifications. For the complex inoculated with enrichment cultures, 1 L of anaerobic mineral medium designated as AGOFS was prepared in a 2 L glass bottle and autoclaved (see supplementary information). For rGO-R4 complex, AGOSF medium was prepared as well. After autoclaving, the medium was mixed with 0.67 g/L of GO and 15 mL of the pre-culture and filled into a glass bottle to remove the headspace. The glass bottle was then incubated at 28 °C for 1 month. After incubation, a hydrogel complex with approximately 30 mm diameter and 10–20 mm height was obtained.

### SEM imaging

For SEM imaging, the rGO-GORBs complex was fixed with 2% glutaraldehyde and 1% osmium tetroxide as described in supporting information. The prepared samples were sputter-coated with gold, and then observed under a field emission scanning electron microscope SU8000 (Hitachi Co., Ltd., Tokyo, Japan) operating at 1.0 kV.

### Electrochemical cultivation

For electrochemical cultivation, a sterilized glass bottle of 0.93 L capacity (90 mm diameter and 175 mm height) connected with a platinum wire as the working electrode was used. The bottle was filled with AGOS medium (see supporting information) to which the rGO-GORB complexes were added. For comparison, GF with 30 mm diameter and 20 mm thickness was used as the representative MFC anode. The GF was immersed in the cell suspension of R4 and the GF holding cells were used as the GF-R4 complex. An Ag/AgCl (KCl salt) electrode and another platinum wire were used as reference and counter electrodes, respectively. The polarization was conducted by setting the working electrode potential at +200 mV v.s. Ag/AgCl by using a potentiostat HA-1510 (Hokuto Denko, Tokyo, Japan). During the polarization, current was recorded every 60 min by using a data logger (T&D Corporation, Nagano, Japan).

### Electrochemical analysis

Cyclic voltammetry (CV) analysis and electrochemical impedance spectroscopy (EIS) of the rGO-GORB complexes were conducted by using an electrochemical measurement system HZ-7000 (Hokuto Denko, Tokyo, Japan). The CV and EIS analyses were performed using the same bottle as that used for electrochemical cultivation described above after 2 h of stabilization with 5.0 mM acetate. CV was conducted at a scan rate of 0.2 mV/s, in the potential range from −400 to 600 mV (v.s. Ag/AgCl). EIS was examined for the rGO-GORB complexes in a frequency range of 100 kHz−0.5 mHz at 200 mV with 20 mV amplitude for the applied ac signal. The Nyquist plots were analyzed using an EIS data analysis software ZSimpWin (Princeton Applied Research, Oak Ridge, TN). CV and EIS analyses were performed at 10d of polarization for the complexes from RW- and PS-cultures while at 20d for the complex from WC-culture. For the complexes with strain R4, data were obtained at 7d and 12d of polarization from the rGO- and GF-complexes of run2 (Supplementary Fig. S2).

### Nucleotide sequence accession numbers

The 16S rRNA gene sequence of strain R4 determined in this study has been deposited under DDBJ accession number LC076693 (http://www.ddbj.nig.ac.jp/Welcome-j.html). A compiled set of the Illumina MiSeq sequences was deposited in Short Read Archive database under accession number DRA003954.

## Additional Information

**How to cite this article**: Yoshida, N. *et al.* Graphene oxide-dependent growth and self-aggregation into a hydrogel complex of exoelectrogenic bacteria. *Sci. Rep.* **6**, 21867; doi: 10.1038/srep21867 (2016).

## Supplementary Material

Supplementary Information

## Figures and Tables

**Table 1:** Abundance of attached and planktonic cells in the three enrichment cultures before and after electrochemical cultivation*.

Culture	Polarization**	Total (×10^9^ cells/culture)	Attached (×10^8^ cells/cm^3^)	Planktonic (×10^7^cells/mL)
RW	–	4.9 ± 1.8	4.7 ± 1.6	1.2 ± 0.58
	+(10 d)	3800 ± 1200	3100 ± 1100	53 ± 4.5
PS	–	4.2 ± 1.0	4.0 ± 2.3	0.60 ± 0.16
	+(10 d)	1300 ± 160	1200 ± 150	17 ± 0.10
WC	–	3.7 ± 1.2	3.4 ± 1.2	0.69 ± 0.23
	+(20 d)	530 ± 61	500 ± 58	32 ± 6.5

^*^The data show the averages and standard deviations of triplicate assays.

^**^Symbols: ^−^before polarization; ^+^polarized.

**Table 2:** Abundance of attached and planktonic cells in R4-culture after 20 d of polarization.

Culture	Total (×10^10^ cells/culture)	Attached (×10^9^ cells/cm^3^)	Planktonic (×10^7^ cells/mL)
rGO-R4	42 ± 5.9	36 ± 5.5	3.3 ± 0.22
GF-R4	6.4 ± 0.66	1.9 ± 0.09	4.1 ± 0.59

^*^The data were obtained from triplicate assays performed in parallel.
