# Supplementary material for: Graphene oxide-dependent growth and self-aggregation into a hydrogel complex of exoelectrogenic bacteria
Source: Sci Rep. 2016 Feb 22;6:21867. doi: 10.1038/srep21867 (PMC4761877; doi:10.1038/srep21867)
Supplement: Supplementary Information [file srep21867-s1.pdf]

Supplementary Information

Graphene oxide-dependent growth and self-aggregation into a hydrogel complex of exoelectrogenic bacteria

Naoko Yoshida, Yasushi Miyata, Kasumi Doi, Yuko Goto, Yuji Nagao, Ryugo Tero, Akira Hiraishi

- **Supplementary Figure S1.** Characterization of GO used in this study.
- **Supplementary Figure S2.** XPS data of C1s spectra in the rGO reduced by strain R4 taken from different three positions.
- **Supplementary Figure S3.** Electric conductivity of 70 mg/L GO (A), the rGO-R4 complex before polarization (B), and biofilm formed on rGO (C).
- **Supplementary Figure S4.** Electricity production by Geobacter sp. R4 using rGO and graphite felt as the anode.
- **Supplementary Figure S5.** Electricity production by *Geobacter* sp. strain R4 using graphite plane as the anode.
- **Supplementary Figure S6.** Electricity production by *Geobacter* sp. strain R4 using GO-coated graphite plane as the anode.
- **Supplementary Figure S7.** CV and EIS data for the complex of strain R4 with rGO or GF before polarization.
- **Supplementary Table S1.** Phylogenetic composition (%) of the three rGO-GORBs complexes after 10-20 d of polarization
- **Supplementary Table S2.** List of primers used in this study
- **Supplementary Table S3.** Abundance of attached and planktonic cells in R4-culture after 50 d of polarization using graphite plane and GO-coated graphite plane.
- **Supplementary methods**

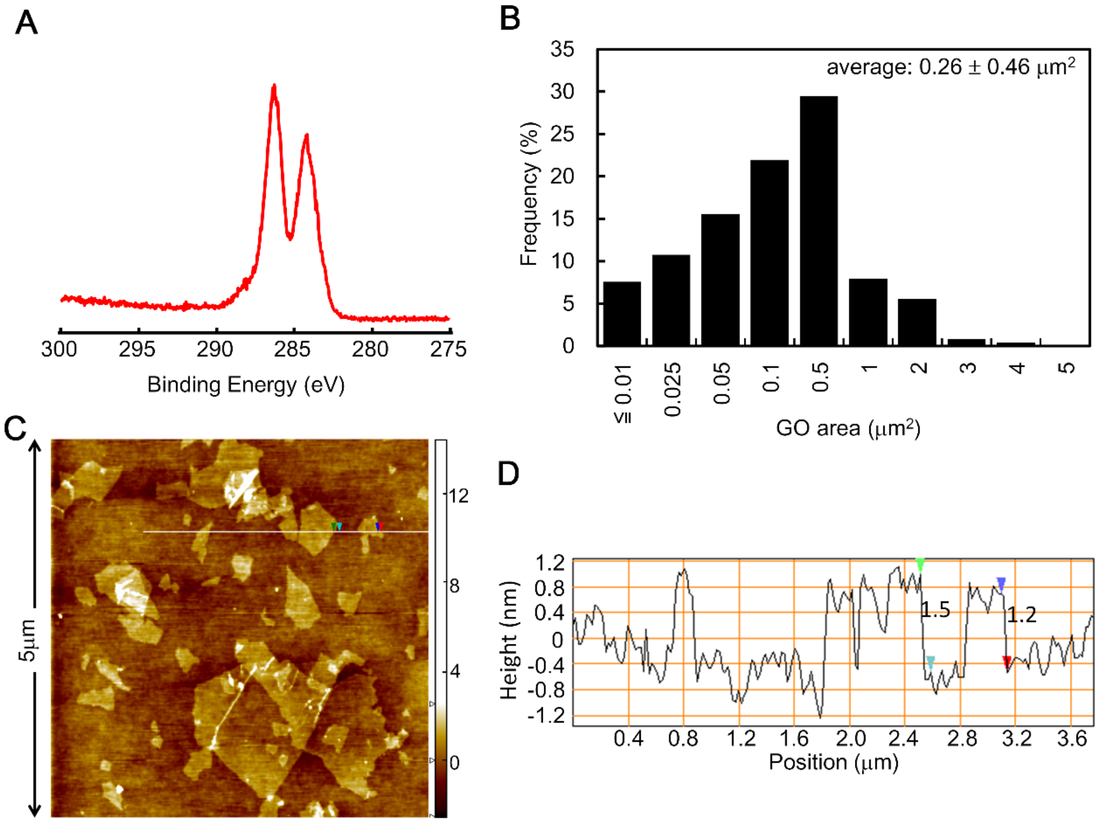

Supplementary Figure S1. Characterization of GO used in this study. (A) XPS spectrum. (B) Histogram of GO area. (C) AFM image. (D) Measurement of GO thickness.

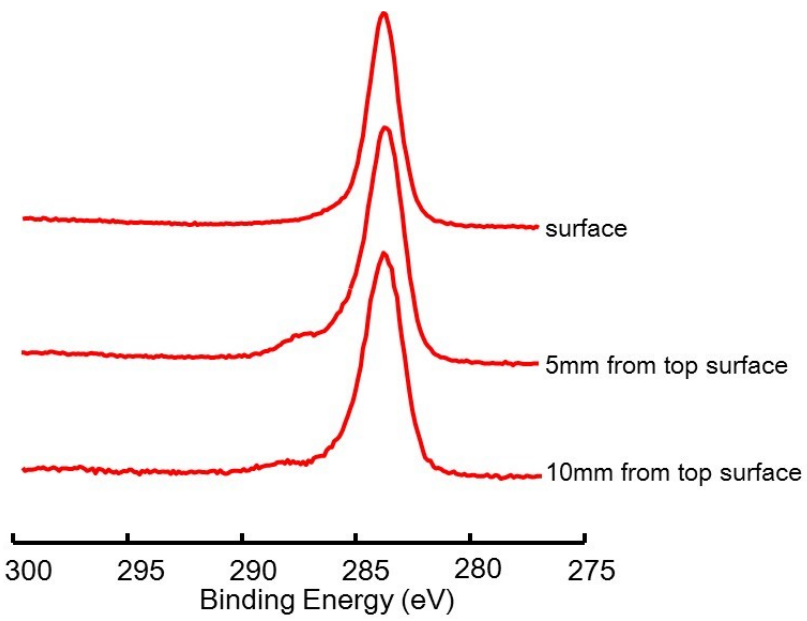

Supplementary Figure S2. XPS data of C1s spectra in the rGO reduced by strain R4 after 36 d of incubation. Three pieces of rGO aggregates were taken from three different parts of the complex, which were at 5 and 10 mm depths from the surface and the core of the complex.

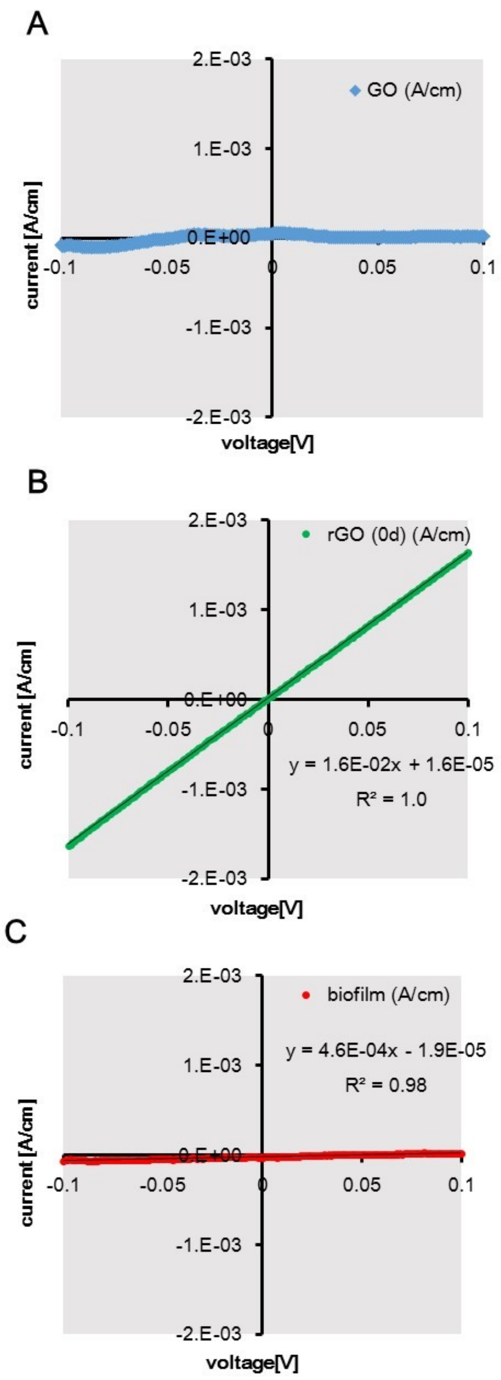

Supplementary Figure S3. Electric conductivity of 70 mg/L GO (A), the rGO-R4 complex before polarization (B), and biofilm formed on rGO (C).

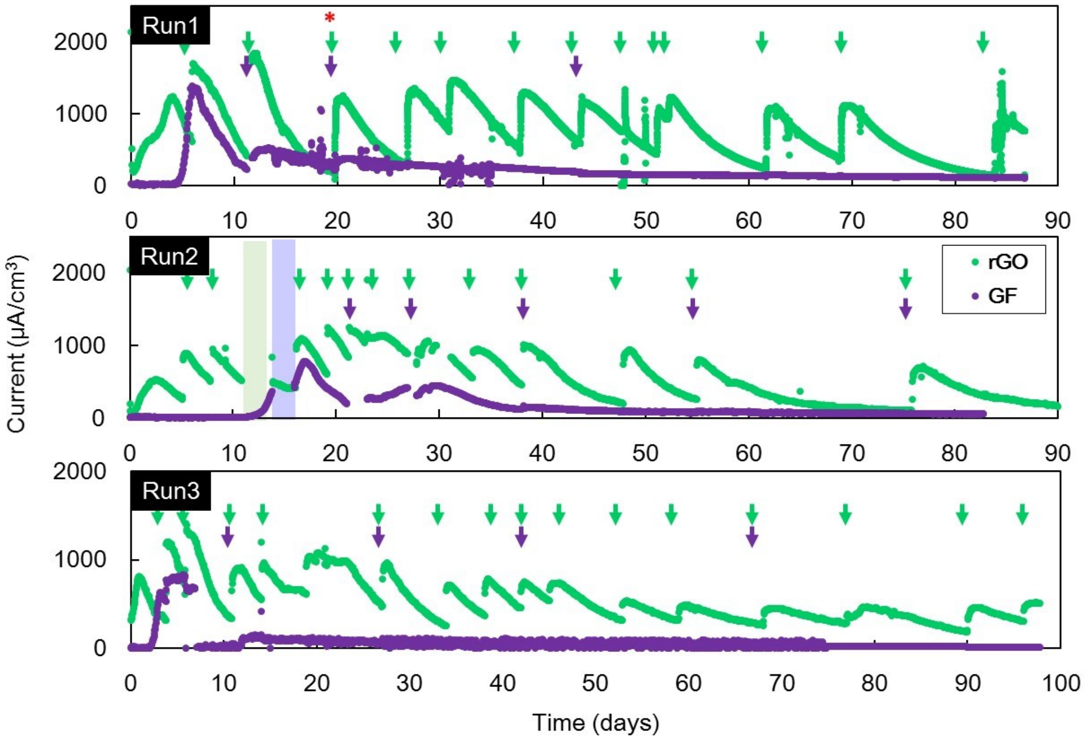

Supplementary Figure S4. Electricity production by *Geobacter* sp. R4 using rGO and graphite felt as the anode. The results of experiments repeated three times are shown as run1-3. The data of run1 was the same data shown in Figure 6A in longer term. Arrows indicate the timing of acetate spiking into the cultures. In run 2, timings of CV and EIS analyses for rGO and GF are highlighted with green and purple, respectively. Red star in run1 indicates the timing of HPLC determination of acetate remained in the cultures.

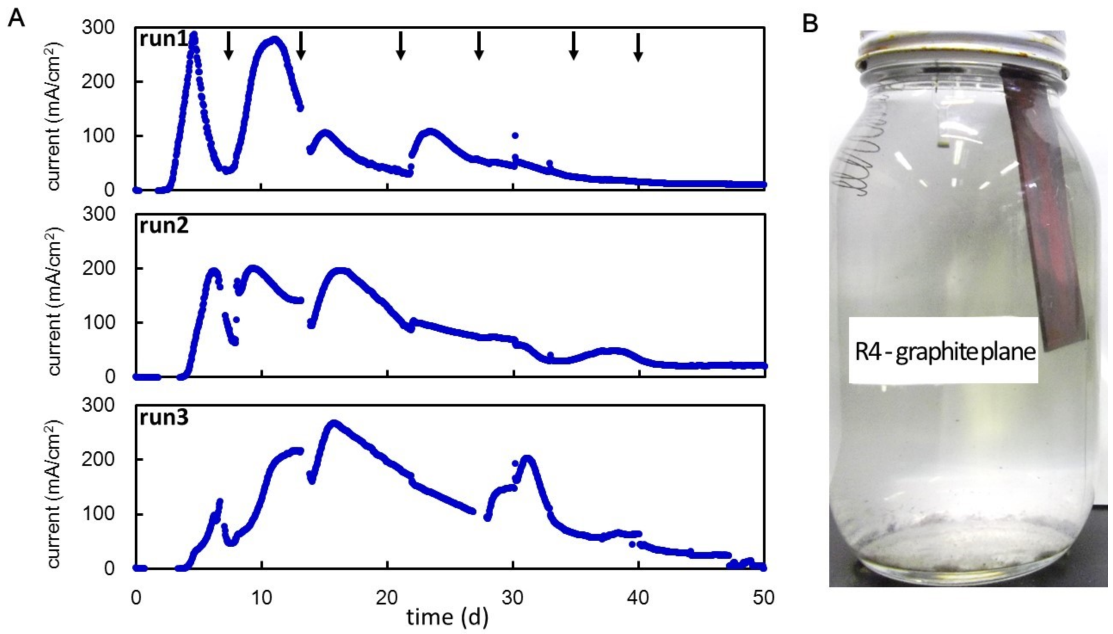

Supplementary Figure S5. Electricity production by *Geobacter* sp. strain R4 using graphite plane as the anode. The results of experiments repeated three times are shown as run1-3

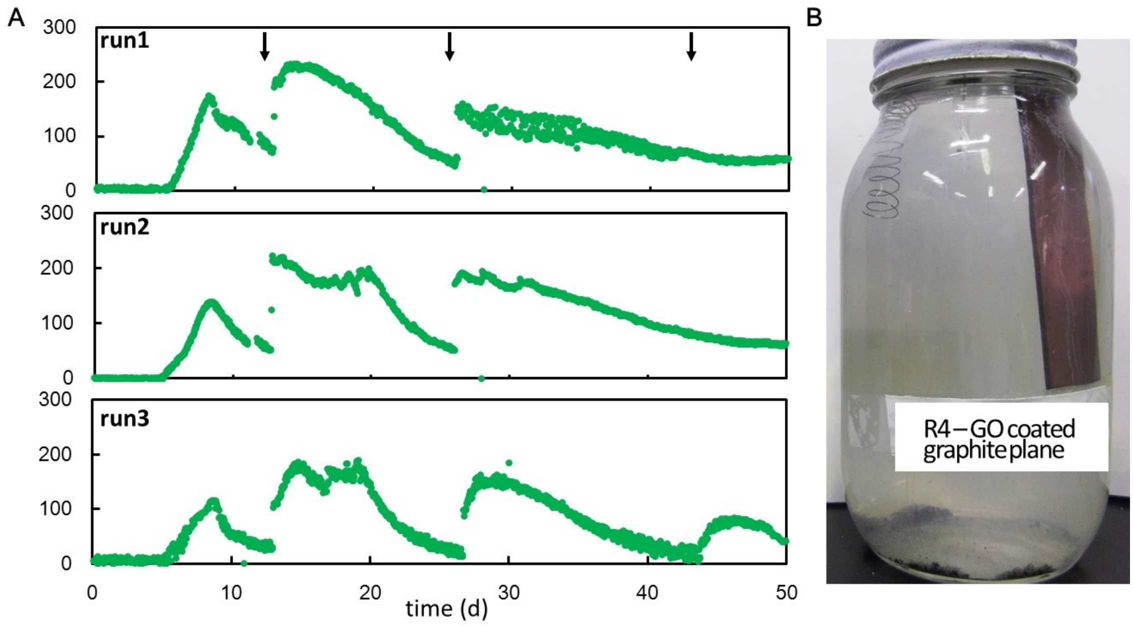

Supplementary Figure S6. Electricity production by *Geobacter* sp. strain R4 using GO-coated graphite plane as the anode. The results of experiments repeated three times are shown as run1-3.

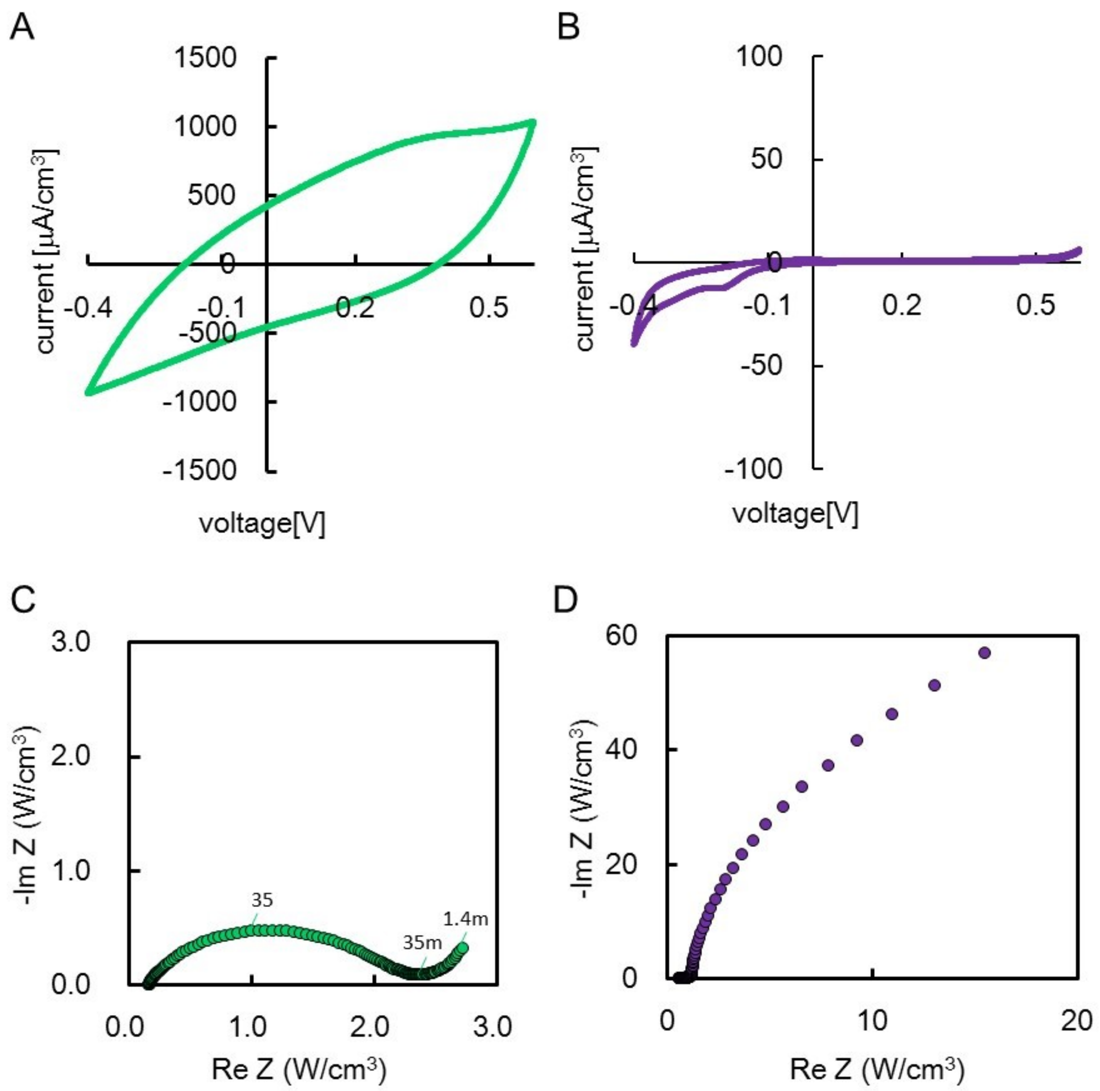

Supplementary Figure S7. CV and EIS data for the complex of strain R4 with rGO or GF before polarization. (A) and (B) show the CV data for the rGO-R4 and GF-R4 complexes, respectively. (C) and (D) indicate the EIS data for the rGO-R4 and GF-R4 complexes, respectively. The numbers on the graph in (C) are frequencies: f [Hz] = $\omega/2\pi$.

Supplementary Table S1. Phylogenetic composition (%) of the three rGO-GORBs complexes after 10-20 d of polarization*

Phylogenetic group	RW	PS	WC
at genus level	+ (10 d)	+ (10 d)	+ (20 d)
Azospira	5.6	7.3	8.6
Geobacter	88	91	77
Oxalobacter	0.35	0.02	<0.01
Sulfurospirillum	0.97	0.04	13
Others	5.5	1.9	1.5

*The data show the results of a simple experiment.

* Symbols: –, before polarization; +, polarized.

Supplementary Table S2. List of primers used in this study

Name	Direction	Sequence (5’- 3’)	Reference
357f	Forward	CCT ACG GGA GGC AGC AG	1
517r	Reverse	ATT ACC GCG GCT GCT GG	1
27f	Forward	AGA GTT TGA TCM TGG CTC AG	2
1492r	Reverse	TACGGYTACCTTGTTACGAC TT	2
515F	Forward	GTG CCA GCM GCC GCG GTA A	3
806R	Reverse	GGA CTA CHV GGG TWT CTA AT	3

1. Muyzer, G., E. C. de Waal, A. G. Uitterlinden. Profiling of complex microbial populations by denaturing gradient gel electrophoresis analysis of polymerase chain reaction-amplified genes coding for 16S rRNA. *Appl. Environ. Microbiol.* **59**, 695-700 (1993).
2. Weisburg, W. G., S. M. Barns, D. A. Pelletier, and D. J. Lane. 16S ribosomal DNA amplification for phylogenetic study. *J. Bacteriol.* **173**:697-703(1991).
3. Caporaso, J. G. *et al.*, Global patterns of 16S rRNA diversity at a depth of millions of sequences per sample. *Proc. Natl. Acad. Sci. U. S. A.* **108**:4516 – 4522 (2011).

Supplementary Table S2. List of primers used in this study

Name	Direction	Sequence (5’- 3’)	Reference
341f	Forward	CCT ACG GGA GGC AGC AG	4
518r	Reverse	ATT ACC GCG GCT GCT GG	4

Supplementary Table S3. Abundance of attached and planktonic cells in R4-culture after 50 d of polarization using graphite plane and GO-coated graphite plane.

	total ($\times 10^{10}$ cells/culture)	attached ($\times 10^{9}$ cells/cm^2)	planktonic ($\times 10^{7}$ cells/mL)
Graphite plane	23 ± 12	3.0 ± 5.8	5.5 ± 2.4
GO-coated graphite plane	28 ±7.4	4.0 ± 3.8	4.2 ± 0.25

* The data were obtained from triplicate assays performed in parallel.

Supplementary methods

Enrichment and isolation of GO reducing bacteria

GO-reducing bacteria (GORBs) were enriched by sequential transfer cultivation of environmental microorganisms using a mineral medium supplemented with GO and acetate as sole electron acceptor and donor, respectively. All procedures were performed anaerobically to avoid contamination of oxygen in the samples as reported previously[1]. We sampled three different inoculums from freshwater environments, i.e. river water (designed as RW, 34°42’22”N, 137°23’31”E), sediment in a water channel (WC, 34°42’26”N, 137°23’42”E), and a paddy soil (PS, 34°42’37”N, 137°23’47”E). Then, 1.0 g-wet wt. of the samples was individually mixed with 1 mL of MilliQ water and the mixture was used as inoculum solutions. Each inoculum solution was introduced to serum bottles with 60 mL capacity including 20 mL of a medium designated as AGOFS.

AGOFS medium was anaerobically prepared as reported previously[2]. AGOFS as the mineral base contained the following components (per Liter): 0.5 g NH_4Cl; 1.0 g NaCl; 0.5 g KCl 0.1 g $CaCl_2 \cdot 2H_2O$; 0.1 g $MgCl_2 \cdot 6H_2O$; 0.2 g KH_2PO_4; 2.5 g $NaHCO_3$; 1 mL of trace element solution SL10; 10 ml of vitamin solution; 1 mL of Se/W solution. The prepared basal medium was then supplemented with 2 mM FeS solution, 10 mM acetate, and 0.67g/L of GO.

The three cultures were incubated at 28 °C for 7 d. At 0 d, GO in all cultures was observed as brown color of GO well dispersed in the whole culture. After 7 d of incubation, the GO changed to black colored aggregates of the reduced form (rGO) as reported previously[3]. Then, the incubated cultures were transferred to fresh medium at 5% of the transfer rate. The cultures were again transferred to fresh medium when the visual change of GO into rGO was observed. The transfer was repeated more than 20 times. The three enriched cultures originally inoculated with river water RW, water channel sediment WC, and paddy soil PS were designed as culture-RW, -WC, and -PS, respectively.

Isolation of GO reducing bacteria

Isolation of GORBs in the three cultures was conducted using an agar plate medium designed as AGOSF-agar. To visualize the capability of GO reduction as black spots of rGO on a plate, 2 mM FeS (black precipitate) in AGOFS was replaced with 1 mM sulfate. The modified medium was designed as AGOSF. The RW culture was serially diluted with 10 mL of 2 fold concentrated AGOSF medium (2×AGOSF) in serum bottles. The series of diluted cultures were then mixed with equal volume of 1% (w/v) Seaplaque GTG agarose (Lonza, Basel, Switzerland). The solidified agar-culture was then placed in a plastic anaerobic jar, Anaeropack jar (Mitsubishi Gas Chemical Co., Tokyo, Japan), with a reducing reagent anaeroPack Anaero puch (Mitsubishi Gas Chemical), and incubated at 28°C for several weeks. All procedures were done in an anaerobic glove box. The colony for which the brown color (indicating GO) changed into black (indicating rGO) was picked from the agar and purified by agar-shake cultivation using AQSY agar medium. AQSY medium is AGOFS medium that has been amended with the addition of 0.5% (w/v) Seaplaque GTG agarose supplemented with 10 mM AQDS instead of 1g/L GO, and to which 0.01% yeast extract has been added. Purification on agar plates was repeated to obtain single uniform colonies as described previously[1]. Isolated strains were usually maintained in AF medium, a modified AGOS medium with supplementation of 10 mM fumarate instead of 1 g/L GO and replacement of 1mM sulfate with 1mM Na_2S.

Illumina MiSeq sequencing of the enriched GORBs and the data analysis

For Illumine-Miseq sequencing, 16SrRNA gene was first amplified using the bacterial and archaeal consensus primers, 515F and 806R (see supporting table). Then the PCR product were tagged by 2nd PCR using commercially provided primers targeting the first primers attached with barcode sequences. The PCR amplicons were purified using a Wizard SV Gel and PCR Clean-Up System (Promega, Fitchburg, WI, USA) and pooled for subsequent pair-end sequencing. Sequencing was performed on Illumina MiSeq platform at the FASMAC Co., Ltd (Atugi, Japan). The sequence reads passed through a

quality filtering using the software sickle ver 1.33 and were trimmed by Fastx toolkit ver 0.0.13.2. The reads filtered with a chimera chimera program, usearch ver 7.0.1090_i86linux64, were analyzed by a microbial community analysis software Qiime ver 1.9.0.

Scanning electron microscope (SEM) imaging

For scanning electron microscope (SEM) imaging, GO and the rGO-GORBs complexes (rGO-RW, -PS, -WC, -R4) were fixed as follows: The rGO-GORBs complexes were washed by immersing in PBS buffer. The washed complexes were resuspended in 2% glutaraldehyde and fixed for 1 h, and then suspended in 1% osmium tetroxide, both at pH 7.2 in PBS buffer. Then, the samples were washed and resuspended in PBS buffer. The fixed samples was gradually dehydrated in acetone and dried. The dried sample was sputter-coated with gold or osmium by Neocoater (JEOL Ltd., Tokyo, Japan) and osmium coater HPC-30 (Vacuum Device Corporation, Ibaragi, Japan), respectively. The coated samples were observed a field emission scanning electron microscope SU8000 (Hitachi Co., Ltd., Tokyo, Japan) operating at 1.0 kV.

Measurement of electric conductivity of GO, the rGO-R4 complex, and biofilm

Electric conductivity was measured by four-terminal sensing using four platinum wires on a glass slide. A sliced sample of the rGO-R4 complex was covered a partition (0.3 mm of thickness, 1 cm^2 of area) having four probes on the surface. Current was measured at voltage in the range from -0.1 to 0.1 mV with 0.5 mV/sec of scan rate. For the analysis of the conductivity of GO, a paste of GO was practically used for the analysis which was obtained in concentration by centrifugation to give approximately equal concentration of GO in the complex. For the analysis of biofilm, biofilm formed on the rGO-R4 complex was physically r collected by a spatula and analyzed.

Electrochemical cultivation of R4 on graphite plane.

To compare the electricity production by strain R4 on different anode fabrication, a graphite plane was additionally assayed for electricity recovery in electrochemical cultivation of R4. The graphite plane was 1mm of thickness and 30 × 100 mm in the size and connected to a platinum wire and used as the working electrode instead of the rGO-R4 complex. To evaluate the effects of GO, the graphite plane was occasionally coated with GO by immersion of the plane in 10 g/L GO stock solution and overnight drying. In the electrical cultivation, strain R4 directly inoculated into the medium to give 8.0×10^8 cells in the culture bottle.

References for supplementary methods

1. Yoshida, N., Ye, L., Liu, F., Li, Z., Katayama, A., Evaluation of biodegradable plastics as solid hydrogen donors for the reductive dechlorination of fthalide by *Dehalobacter* species. *Biores. Technol.* **2013**, *130*, 478-485.
2. Yoshida, N., Ye, L., Baba, D., Katayama, A., A Novel *Dehalobacter* Species Is Involved in Extensive 4,5,6,7-Tetrachlorophthalide Dechlorination. *Appl. Environ. Microbiol.* **2009**, *75* (8), 2400-2405.
3. Straub, K. L., Benz, M., Schink, B., Widdel, F., Anaerobic, nitrate-dependent microbial oxidation of ferrous iron. *Appl. Environ. Microbiol.* **1996**, *62* (4), 1458-1460.
4. Tanizawa, Y., Okamoto, Y., Tsuzuki, K., Nagao, Y., Yoshida, N., Tero, R., Iwasa, S., Hiraishi, A., Suda, Y., Takikawa, H. *Microorganism mediated synthesis of reduced graphene oxide films*, *J. Phys. Conf. Ser.* **2012**, *352,* 012011.
